# Anti-reflux mucosal ablation (ARMA) as a novel minimally invasive treatment for severe gastroesophageal reflux disease in a child: the first pediatric case report

**DOI:** 10.3389/fped.2026.1812619

**Published:** 2026-06-01

**Authors:** Jonas Povilavičius, Geistė Tubutytė, Kamilė Bagdonaitė, Audrius Dulskas, Austėja Račytė, Arūnas Strumila

**Affiliations:** Vilnius University Faculty of Medicine, Vilnius, Lithuania

**Keywords:** anti-reflux mucosal ablation, endoscopic therapy, gastroesophageal reflux disease, neurological impairment, pediatrics

## Abstract

**Background:**

Antireflux mucosal ablation (ARMA) is a novel, minimally invasive endoscopic technique for the treatment of gastroesophageal reflux disease (GERD). Our case report evaluates the feasibility and early clinical outcomes of ARMA in a neurologically impaired pediatric patient with refractory GERD.

**Case description:**

A 9-year-old boy with spastic cerebral palsy presented with severe GERD refractory to a long-term proton pump inhibitor therapy. He suffered from profound oropharyngeal dysphagia and recurrent aspiration pneumonia requiring multiple hospitalization. An endoscopic ARMA procedure was performed under general anesthesia without intra-procedural complications or immediate adverse events. Early follow-up revealed improved reflux control, enhanced tolerance of gastrostomy feeding, and a reduced need for acid-suppressive therapy. No respiratory deterioration or procedure-related complications were observed.

**Conclusions:**

ARMA is a technically feasible, minimally invasive option for refractory GERD. While this initial case involved a high-risk, neurologically impaired child, the mechanism suggests broader applicability to other pediatric patients with medication-resistant GERD.

## Introduction

Neurologically impaired children face a higher risk of GERD, making effective treatment vital for improving their quality of life (1). Although proton pump inhibitors (PPIs) are the first-line treatment, nearly **40%** of patients experience persistent symptoms despite therapy (2). For those with refractory GERD—particularly neurologically impaired children—therapeutic options are limited, as conventional surgery carries significant procedural risks and morbidity. Consequently, there is growing interest in minimally invasive endoscopic alternatives (3). ARMA represents a novel technique not previously reported in the pediatric population (4).

## Case description

As this report describes a single case, no formal allocation or randomization was applicable. Due to his high surgical risk and comorbidities, a multidisciplinary team preferred a minimally invasive endoscopic approach over conventional fundoplication. The patient was selected due to severe, medication-refractory GERD (Los Angeles Grade C) in the context of significant neurological impairment and high surgical risk.

Conventional surgical treatment (fundoplication) was considered. However, due to the patient's comorbidities and an increased risk of perioperative complications, a minimally invasive endoscopic approach was preferred. This case therefore represents a highly selected clinical scenario, and the potential for selection bias must be acknowledged.

A 9-year-old boy with spastic cerebral palsy presented with severe GERD refractory to long-term PPI therapy. The patient was previously diagnosed with severe oropharyngeal dysphagia with a high risk of aspiration and was fully dependent on gastrostomy feeding. He also experienced recurrent aspiration pneumonia requiring multiple hospitalization. Aspiration was therefore considered multifactorial, with both impaired airway protection and severe GERD contributing. The diagnostic process was complicated by the presence of neurological impairment, which contributed to a multifactorial risk of aspiration. Differentiating the relative contribution of these factors represented a key diagnostic challenge.

The differential diagnosis included primary aspiration secondary to neurogenic dysphagia without significant reflux, gastroparesis-related regurgitation, and non-acid reflux. However, the severity of endoscopic findings (Los Angeles grade C esophagitis and Hill grade III valve) strongly supported GERD as a major contributing factor.

Given the persistence of symptoms despite prolonged PPI therapy, the condition was classified as refractory GERD. Prognostically, the patient was a high-risk due to the neurological impairment, which is associated with an increased risk of treatment failure, recurrent reflux, and complications following conventional surgical interventions.

Overall, the integration of clinical, endoscopic, and functional considerations guided the decision-making process and supported selection of a minimally invasive endoscopic approach.

**Figure 1:**
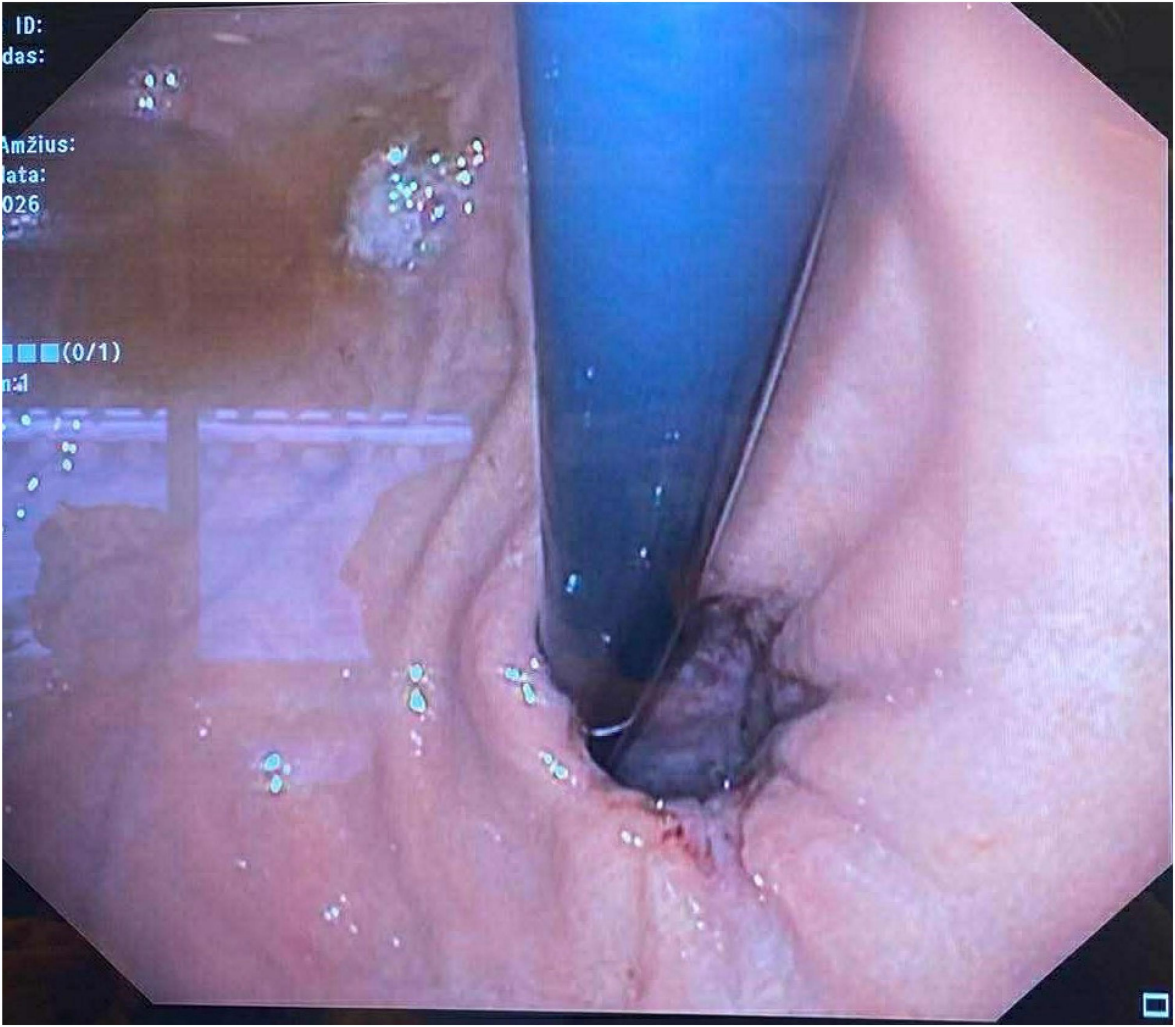
Index endoscopy demonstrating a gastroesophageal (GE) flap valve, hill grade III.

**Figure 2:**
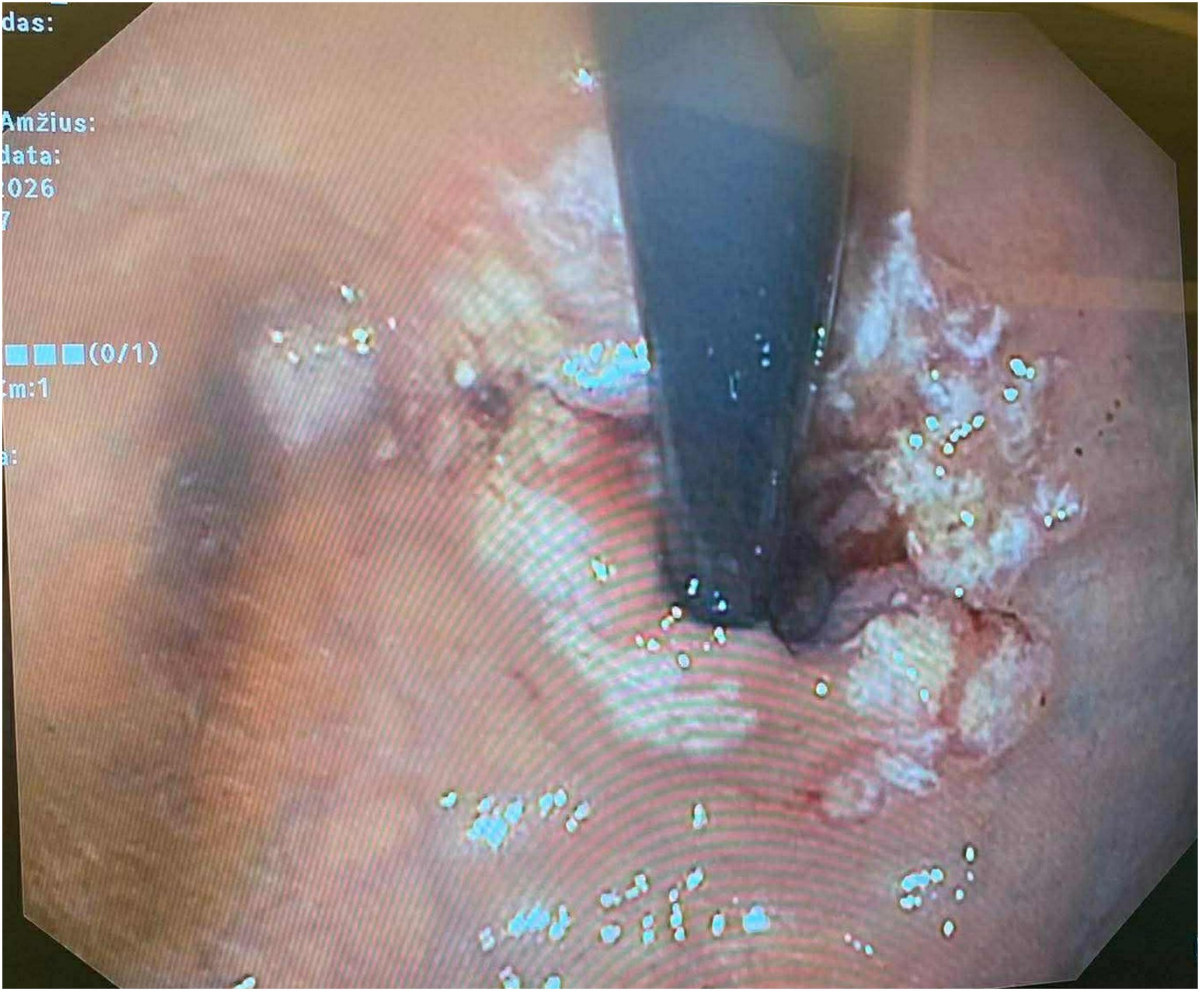
Anti-reflux mucosal ablation (ARMA) procedure.

**Figure 3:**
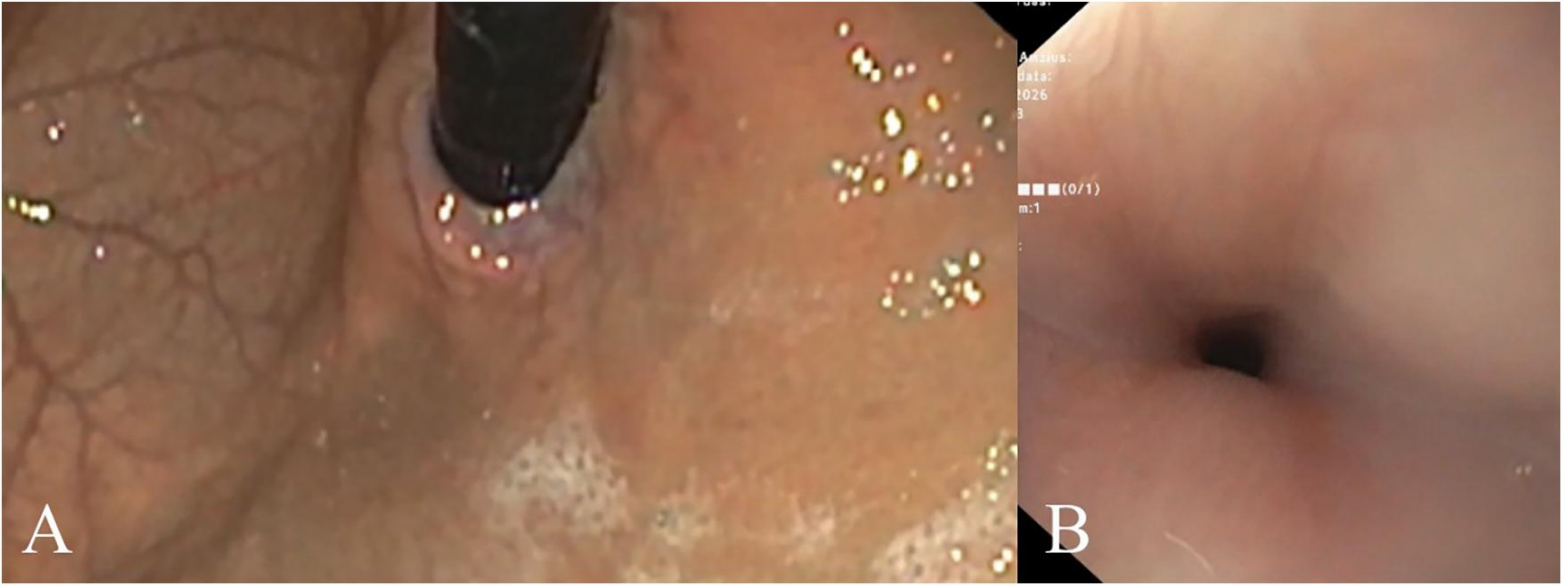
One-month follow-up endoscopy revealing marked improvement of the GE flap valve to hill grade 0 **(A)** and post-procedurally narrowed cardia **(B).**

Given his significant comorbidities, conventional antireflux surgery was considered high risk. Endoscopic ARMA was performed under general anesthesia. The index endoscopy demonstrated a gastroesophageal (GE) flap valve graded as Hill grade III (Figure 1). A circumferential targeted mucosal ablation (Figure 2) was applied at the gastroesophageal junction with the objective of inducing controlled mucosal contraction and fibrosis to reinforce the antireflux barrier. The procedure was technically successful and completed without intraprocedural complications. No immediate adverse events occurred, and post-procedural recovery was uneventful. At one-month follow-up, control endoscopy demonstrated a marked improvement of the GE flap valve to Hill grade 0 (Figure 3). Parental interviews revealed a significant reduction in GERD symptoms, improved tolerance of enteral nutrition via gastrostomy, and decreased reliance on acid-suppressive therapy. No early respiratory deterioration or procedure-related complications were observed.

## Discussion

ARMA is an emerging endoscopic therapy that uses targeted mucosal ablation at the gastric cardia to induce scarring and reshape the mucosal flap valve, enhancing its anti-reflux barrier without the need for full-thickness fundoplication (2, 4). Initially described in adults with PPI-refractory GERD, ARMA has shown promising results in improving GERD-related symptoms and objective acid-reflux parameters, with an acceptable safety profile (2). To our knowledge, this is the first report describing the application of ARMA in a neurologically impaired child with severe, medication-refractory GERD who was considered high risk for conventional surgery, thereby extending the potential indication of this technique to a highly vulnerable population.

In adult cohorts, ARMA has been associated with significant improvement in validated GERD symptom scores and objective pH-metric parameters, with clinical success rates of approximately 60%–70% at 6–12 months (2, 5). Shimamura et al., in a bi-institutional study of 68 adults, reported sustained symptom improvement at one year and demonstrated that ARMA is technically feasible and reproducible, with most procedures completed without major adverse events (5). Previous studies have demonstrated that the mean Hill flap valve grade improves from lax Grade III toward more competent Grades I–II following intervention, indicating restoration of gastroesophageal junction valve competence (6). In our patient, the observed endoscopic transition from Hill grade III to Hill grade 0 at one-month follow-up, accompanied by a clear reduction in clinical reflux symptoms and decreased reliance on acid-suppressive therapy, mirrors these adult findings and supports the mechanistic rationale for ARMA in modulating the GEJ barrier even in the pediatric setting.

An important further limitation is the short follow-up period of one month, which is insufficient to reliably assess long-term symptom control, recurrence rates, or delayed complications such as oesophageal stenosis. Early clinical improvement should therefore be interpreted with caution, and longer-term follow-up is essential to confirm durability of the therapeutic effect.

Antireflux surgery—particularly fundoplication—is an established and generally effective treatment for pediatric GERD, with good outcomes in appropriately selected patients. Indeed, several studies report high rates of symptom control (often >80%–85%) and acceptable morbidity in the general pediatric population (7). Our statement specifically refers to neurologically impaired children, who represent a distinct and more vulnerable subgroup. In this population, the risk profile and long-term outcomes differ significantly from neurologically normal children. Children with severe neurological impairment represent a distinct high-risk GERD population in whom conventional antireflux surgery, particularly Nissen fundoplication, is associated with a higher incidence of postoperative complications such as postoperative pneumonia, retching, bowel obstruction, and even early mortality, and a considerable proportion of patients experience recurrent reflux or require reoperation (8). Recurrence rates after fundoplication are higher in neurologically impaired children (approximately 12%–30% vs. 2%–2.6% in neurologically normal children) (9). Failure rates may reach 20%–40%, with increased need for reintervention (7). Major complication rates up to ∼29% have been reported in comparative analyses (10). Mortality, although generally low, is higher in neurologically impaired patients (reported up to ∼3% or higher in selected cohorts) (7). Additionally, perioperative complications (e.g., respiratory events, dysphagia, gas-bloat syndrome) and persistent symptoms are more frequent in this group (7). Importantly, while surgery often improves symptoms and reduces hospitalizations, these benefits must be interpreted in the context of substantial comorbidity burden, aspiration risk, and overall frailty, which contribute to higher perioperative and long-term risk. In this context, a minimally invasive endoscopic therapy that can be performed in a relatively short procedure and is potentially repeatable offers an attractive alternative strategy for children in whom surgical risk is prohibitive or in whom caregivers prefer to defer or avoid fundoplication.

Our case highlights several practical advantages of ARMA in a neurologically impaired child with severe, refractory GERD. First, the procedure was technically straightforward and completed without intra-procedural or early post-procedural complications, consistent with adult series that describe ARMA as relatively simple and widely accessible using standard therapeutic endoscopic equipment (2, 5). Second, symptom improvement occurred rapidly and was accompanied by objective endoscopic improvement of the gastroesophageal flap valve, aligning with reports that ARMA effectively narrows the cardia and restores anti-reflux function (2, 4, 5). Third, in this patient with recurrent aspiration pneumonia and high operative risk, ARMA provided a minimally invasive option that reduced reflux-related symptoms and improved feeding tolerance without exposing the child to the full perioperative risk profile of fundoplication. These findings suggest that ARMA may be particularly valuable for neurologically impaired children who are poor surgical candidates, as a definitive therapy, a temporizing measure, or a step in a staged management pathway.

Despite these encouraging observations, the limitations of this report and of the current evidence must be acknowledged. Experience with ARMA remains largely confined to adult cohorts, and long-term durability beyond one to two years is still being defined, with some patients requiring repeat procedures or additional therapies. Pediatric data are absent, and the safety profile, optimal lesion design, and appropriate patient selection criteria in children—especially those with complex comorbidities—are unknown. Potential adverse events such as post-procedural stenosis, transient dysphagia, or chest pain have been described in adults, including a need for endoscopic dilation in a subset of patients, and might be more consequential in small children with limited reserve. Our follow-up period was short, and we did not perform objective pH-impedance monitoring after ARMA, which limits conclusions about long-term acid exposure and the durability of symptom relief. Consequently, ARMA in pediatrics should presently be regarded as an investigational, off-label option that must be considered only after multidisciplinary discussion, detailed counseling of caregivers, and careful weighing of risks and benefits in comparison with established surgical and medical approaches.

Given the single-case design, no statistical analysis or power calculation was applicable. Consequently, the present findings cannot be generalized, and rare adverse events or complications cannot be adequately assessed. While the observed improvement in symptoms and endoscopic findings is clinically meaningful, no statistical inference can be made. The results should therefore be interpreted as indicative of potential clinical benefit rather than evidence of efficacy.

## Conclusions

ARMA appears to be a technically feasible and minimally invasive treatment option for refractory GERD in a highly selected case. However, its efficacy and safety in the paediatric population remain to be established. Although this first pediatric case was performed in a neurologically impaired child with high surgical risk, the mechanism of ARMA suggests that this approach may be applicable more broadly to children with medication-resistant GERD, including those without neurologic impairment. Further prospective studies are warranted to define standardized ARMA protocols, evaluate long-term efficacy, durability, and safety in the pediatric population.

## Future directions

Further research is required to define the role of ARMA in the management of paediatric GERD. Prospective studies with larger patient cohorts are needed to evaluate the efficacy, safety, and durability of ARMA in children, including both neurologically impaired and neurologically normal populations.

Future investigations should incorporate objective outcome measures, such as pH-impedance monitoring and validated symptom scoring systems, to better quantify treatment response and correlate clinical improvement with physiological changes in reflux burden. Standardisation of procedural parameters—including energy settings, extent and pattern of mucosal ablation, and optimal patient selection criteria—will be essential to ensure reproducibility and comparability across centres.

Long-term follow-up studies are particularly important to assess durability of effect, recurrence rates, and late adverse events, including potential oesophageal stenosis or functional complications. Comparative studies evaluating ARMA against established interventions, such as fundoplication or other endoscopic anti-reflux techniques, would further clarify its position within the therapeutic algorithm.

Finally, given the minimally invasive and potentially repeatable nature of ARMA, future research should explore its role not only as a definitive treatment, but also as a bridging or adjunctive therapy in staged management strategies for complex paediatric GERD.

## Limitations

This report has several limitations that should be considered when interpreting the findings. First, it describes a single case, which inherently limits generalisability and precludes any firm conclusions regarding efficacy, safety, or reproducibility of ARMA in the paediatric population. Individual patient characteristics—particularly the presence of severe neurological impairment—may have influenced both the clinical presentation and the observed response to treatment.

Second, the follow-up period was short, restricting the ability to assess long-term durability of symptom control, recurrence of gastroesophageal reflux, and the potential development of delayed adverse events such as oesophageal stenosis or dysphagia. Longer follow-up with repeated clinical and endoscopic evaluation is necessary to determine sustained efficacy.

Third, objective assessment of reflux control following ARMA was limited. We did not perform post-procedural pH-impedance monitoring or quantify acid exposure, relying instead on endoscopic findings and caregiver-reported clinical improvement. Although these outcomes are clinically meaningful, they may be subject to bias and do not fully capture physiological changes in reflux burden.

Fourth, this case involved a neurologically impaired child with complex comorbidities, including severe oropharyngeal dysphagia and aspiration risk. As a result, symptom improvement may reflect a combination of factors, and the specific contribution of ARMA to the reduction of aspiration events cannot be definitively isolated.

Finally, ARMA remains an emerging and off-label technique in paediatric patients. There are currently no standardized protocols regarding energy settings, extent of mucosal ablation, or patient selection criteria in children. This lack of standardization may affect reproducibility and highlights the need for prospective studies and consensus guidelines.

Ultimately, this report represents a single-patient experience, which precludes any statistical analysis and limits external validity. The findings should therefore be interpreted as hypothesis-generating rather than confirmatory, and no conclusions regarding comparative effectiveness versus standard surgical treatment can be drawn.

## Patient perspective

The patient’s caregivers reported marked improvement in feeding tolerance and reduction in respiratory events, describing a significant improvement in quality of life.

## Data Availability

The raw data supporting the conclusions of this article will be made available by the authors, without undue reservation.

## References

[1] Dewan T Turner J Lethebe BC Johnson DW. Gastro-oesophageal reflux disease in children with neurological impairment: a retrospective cohort study. BMJ Paediatr Open. (2022) 6(1):e001577. 10.1136/bmjpo-2022-001577 36645746 PMC9490596

[2] Inoue H Tanabe M de Santiago ER Abad MRA Shimamura Y Fujiyoshi Y. Anti-reflux mucosal ablation (ARMA) as a new treatment for gastroesophageal reflux refractory to proton pump inhibitors: a pilot study. Endosc Int Open. (2020) 8(2):E133–8. 10.1055/a-1031-9436 32010745 PMC6976329

[3] Nabi Z Reddy DN. Update on endoscopic approaches for the management of gastroesophageal reflux disease. Gastroenterol Hepatol (N Y). (2019) 15(7):369–76. 31391807 PMC6676348

[4] Tanabe M Inoue H. Anti-reflux mucosal ablation (ARMA). In: Ujiki M Callahan ZM, editors. Third Space Endoscopy. Cham, Switzerland: Springer Nature Switzerland (2025). p. 73–81. 10.1007/978-3-031-84509-3_7

[5] Shimamura Y Inoue H Tanabe M Ushikubo K Yamamoto K Kimoto Y. Clinical outcomes of anti-reflux mucosal ablation for gastroesophageal reflux disease: an international bi-institutional study. J Gastroenterol Hepatol. (2024) 39(1):149–56. 10.1111/jgh.16370 37787176

[6] Sharma Z Sisodia S Choudhary N Bansal R Choudhuri G. Endoscopic mucosal techniques for GERD: dawn of new era in endoscopic GERD management. J Dig Endosc. (2025) 16(2):068–76. 10.1055/s-0045-1809363

[7] Flores JC Campos JM Cohen E Torres-Robles R Atenafu EG Arredondo C. Gastrostomy plus fundoplication or gastro-jejunal tube versus gastrostomy alone for gastro-esophageal reflux in children with neurological impairment. Cochrane Database Syst Rev. (2022) 2022(11):CD015007. 10.1002/14651858.CD015007 PMC13188193 42159156

[8] Chao NSY Leung MWY Poon M Wong BPY Chung KW Kwok WK. Fundoplication in children with neurological impairment: a worthwhile surgical treatment? Hong Kong J Paediatr (New Series). (2009) 14:152–7. Available online at: https://www.hkjpaed.org/pdf/2009;14;152-157.pdf (Accessed January 28, 2026).

[9] Ichinose A Konishi KI Takazawa S Sunouchi T Suzuki K Yoshida M. Risk factors for recurrence of gastroesophageal reflux disease after laparoscopic nissen fundoplication in patients with severe motor and intellectual disabilities. Asian J Endosc Surg. (2025) 18(1):e70085. 10.1111/ases.70085 40425463 PMC12116254

[10] Livingston MH Shawyer AC Rosenbaum PL Jones SA Walton JM. Fundoplication and gastrostomy versus percutaneous gastrojejunostomy for gastroesophageal reflux in children with neurologic impairment: a systematic review and meta-analysis. J Pediatr Surg. (2015) 50(5):707–14. 10.1016/j.jpedsurg.2015.02.020 25783384

