# Correction: Anti-reflux mucosal ablation (ARMA) as a novel minimally invasive treatment for severe gastroesophageal reflux disease in a child: the first pediatric case report

**DOI:** 10.3389/fped.2026.1909613

**Published:** 2026-07-22

**Authors:** Jonas Povilavičius, Geistė Tubutytė, Kamilė Bagdonaitė, Audrius Dulskas, Austėja Račytė, Arūnas Strumila

**Affiliations:** Vilnius University Faculty of Medicine, Vilnius, Lithuania

**Keywords:** anti-reflux mucosal ablation, endoscopic therapy, gastroesophageal reflux disease, neurological impairment, pediatrics

**Author affiliation**

Authors Jonas Povilavičius, Geistė Tubutytė, Kamilė Bagdonaitė, Arūnas Strumila and Austėja Račytė were erroneously assigned to affiliation “Vilnius University Hospital Santaros Klinikos, Children's Hospital, Paediatric Surgery Department, Vilnius, Lithuania”. This affiliation has now been removed for these authors.

Author Audrius Dulskas was erroneously assigned to affiliation “National Cancer Institute, Vilnius, Lithuania”. This affiliation has now been removed for author Audrius Dulskas.

**Error in author list**

The order of authors in the author list of the published paper was erroneously given as:

Jonas Povilavičius, Geistė Tubutytė, Kamilė Bagdonaitė, Audrius Dulskas, Arūnas Strumila, Austėja Račytė

The correct author list reads:

Jonas Povilavičius, Geistė Tubutytė, Kamilė Bagdonaitė, Audrius Dulskas, Austėja Račytė, Arūnas Strumila

The original version of this article has been updated.

